# Changes of hospitalization trend in the pediatric cardiology division of a single center by increasing adult with congenital heart disease

**DOI:** 10.1186/s12872-020-01511-3

**Published:** 2020-05-15

**Authors:** Sang-Yun Lee, Gi-Beom Kim, Hye-Won Kwon, Mi-kyoung Song, Eun Jung Bae, Sungkyu Cho, Jae Gun Kwak, Hong-Gook Lim, Woong-Han Kim, Jeong-Ryul Lee

**Affiliations:** 1 Department of Pediatrics, Seoul National University Children’s Hospital, Seoul National University College of Medicine, 101, Daehak-ro, Jongno-gu, Seoul, 03080 Republic of Korea; 2 Department of Thoracic and Cardiovascular Surgery, Seoul National University Children’s Hospital, Seoul National University College of Medicine, Seoul, Korea

**Keywords:** Adult with congenital heart disease

## Abstract

**Background:**

As a result of advances in pediatric care and diagnostic testing, there is a growing population of adults with congenital heart disease (ACHD). The purpose of this study was to better define the epidemiology and changes in the trend of hospitalizations for ACHD in Korean society.

**Methods:**

We reviewed outpatient and inpatient data from 2005 to 2017 to identify patient ≥18 years of age admitted for acute care with a congenital heart disease (CHD) diagnosis in the pediatric cardiology division. We tried to analyze changes of hospitalization trend for ACHD.

**Results:**

The ratio of outpatients with ACHD increased 286.5%, from 11.1% (1748/15,682) in 2005 to 31.8% (7795/24,532) in 2017. The number of ACHD hospitalizations increased 360.7%, from 8.9% (37/414) in 2005 to 32.1% (226/705) in 2017. The average patient age increased from 24.3 years in 2005 to 27.4 in 2017. The main diagnosis for admission of ACHD is heart failure, arrhythmia and Fontan-related complications. The annual ICU admission percentage was around 5% and mean length of intensive care unit (ICU) stay was 8.4 ± 14.6 days. Mean personal hospital charges by admission of ACHD increased to around two times from 2005 to 2017. (from $2578.1 to $3697.0). Total annual hospital charges by ACHD markedly increased ten times (from $95,389.7 to $831,834.2).

**Conclusions:**

The number of hospital cares for ACHD dramatically increased more than five times from 2005 to 2017. We need preparations for efficient healthcare for adults with CHD such as a multi-dimensional approach, effective communication, and professional training.

## Background

Congenital heart disease (CHD) is the most common congenital lesion, and the overall prevalence is known to be 0.8 ~ 0.9% [1]. According to a report by the Korea Heart Foundation, around 5000 patients undergo treatment for CHD annually in South Korea, and their mortality is around 2% (http://www.heart.or.kr). The incidence of adults living with CHD has been increasing every year due to remarkable advances in surgically or percutaneously interventional techniques and devices and knowledge of critical care for patients with CHD [2]. The majority of these adult patients cannot be considered to be completely cured, as most lesions have a chronic course, even after repair. Also, patients who have undergone cardiac surgery are more likely to have vulnerable heart and systemic conditions [3, 4].

In Kim’s report [5], he expected that there will be about 70,000 patients in South Korea by the year 2020. The increase in adult CHD patients in South Korea is also a global trend in the developed countries, and special programs for them have been reported in many societies [6, 7]. In this study, we intended to investigate epidemiologic changes of patient population in the pediatric cardiology division by analyzing changes of hospitalization trend for adults with CHD in the pediatric cardiology division of a single tertiary center in South Korea.

## Methods

We reviewed the medical records of outpatients and inpatients from 2005 to 2017 in the pediatric cardiology division of Seoul National University Children’s Hospital. We identified adult patients (≥ 18 years old) with a CHD. We analyzed change of hospitalization trend for adults with CHD.

Demographic covariates included age, gender, and year of admission. Each diagnosis was categorized as simple, complex, or unclassified based on the 32nd Bethesda Conference report [3]. According to the 32nd Bethesda Conference report, types of adult patients with simple congenital heart diseases were classified as simple lesions and types of adult patients with congenital heart disease moderate or great severity were classified as complex lesions. Diseases not mentioned in the 32nd Bethesda Conference report were classified as unclassified. The diagnosis classification of 32nd Bethesda Conference report is described in the supplement table.

Diagnoses classified as moderately or severely complex in the 32nd Bethesda Conference document are defined as complex in this analysis. Patients with isolated simple defects and coexisting pulmonary hypertension were categorized as complex. We defined comorbidities based on Elixhauser’s comprehensive set of comorbidities [8].

Non-CHD diagnoses were defined by the following International Classification of Diseases (ICD)-10 diagnosis codes: cardiomyopathy, electrophysiology diagnoses, ischemic stroke or transient ischemic attack, soft tissue infection, and pregnancy. Procedures were defined by the following ICD-10 procedure codes: percutaneous patent ductus arteriosus (PDA) or atrial septal defect/ patent foramen ovale (ASD/PFO) closure, implantable cardioverter-defibrillator insertion or revision, pacemaker insertion or revision, and percutaneous coronary intervention.

Primary outcomes of interest were the annual number of visiting the outpatient clinic and admission with all CHD diagnoses, as well as annual admissions for subgroups of the overall population such as simple and complex CHD diagnoses. Secondary outcomes of interest were the frequency of specific diagnoses, procedures associated with hospitalizations for ACHD, hospitalization duration, and hospital charges.

This study used clinical data retrieved from the Seoul National University Hospital Patients Research Environment (SUPREME) system. This study was approved by the institutional review board of Seoul National University Hospital (Approved date: Dec 14, 2018 / IRB number: 1811–059-983) and patient’s consent was waived for retrospective study nature.

## Results

**Fig. 1:**
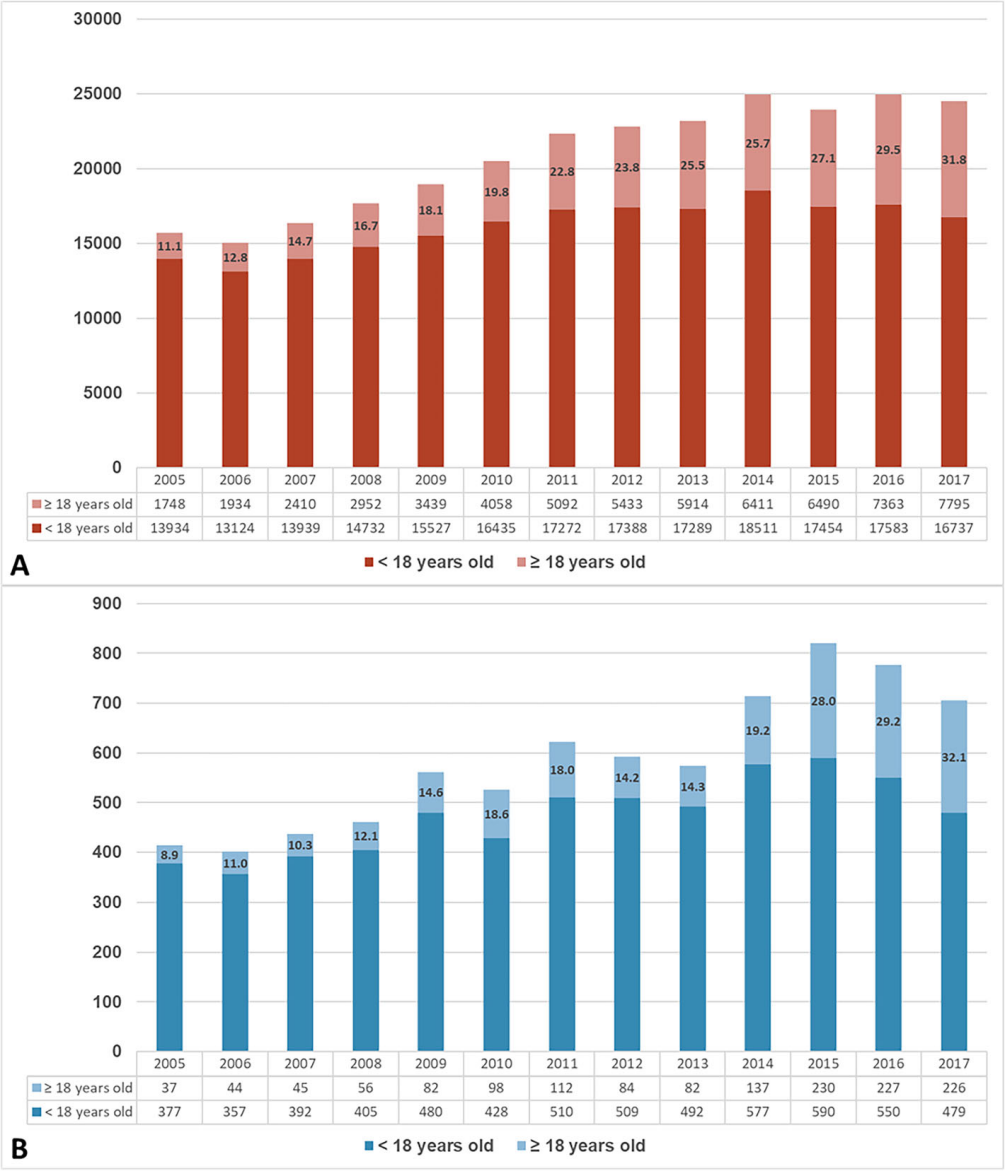
**a** Annual number of out-patient clinic and (**b**) annual number of admission patients in the pediatric cardiology division. The ratio of outpatients with ACHD increased 286.5%, from 11.1% (1748/15,682) in 2005 to 31.8% (7795/24,532) in 2017. The number of ACHD hospitalizations increased 360.7%, from 8.9% (37/414) in 2005 to 32.1% (226/705) in 2017

We analyzed the annual number of outpatient clinic visits in the pediatric cardiology division of our hospital, and the ratio of adult patients increased from 11.1 to 31.8% from 2005 to 2017 (Fig. 1). During this period, children patients increased 20.1% (from 13,934 to 16,737 a year) and adult patients increased 445.9% (from 1748 to 7795 a year) in the outpatient clinic. In analyzing the annual admission number, the ratio increased from 8.9 to 32.1% from 2005 to 2017 (Fig. 1b). During this period, the number of children patients admitted increased 27.1% (from 377 to 479 a year) and adult patients increased 610.8% (from 37 to 226 a year).

**Fig. 2:**
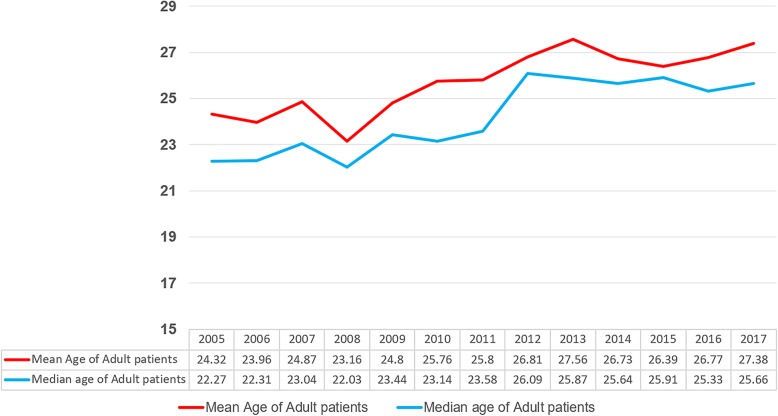
Age of Adult patients at admission in the pediatric cardiology division. The mean age of adult patients at admission increased from 24.3 to 27.4 years

The mean age of adult patients at admission increased from 24.3 to 27.4 years (Fig. 2). The absolute number of adult patients with CHD in the outpatient clinic and admissions increased around 6.1 times from 2005 to 2017.

**Fig. 3:**
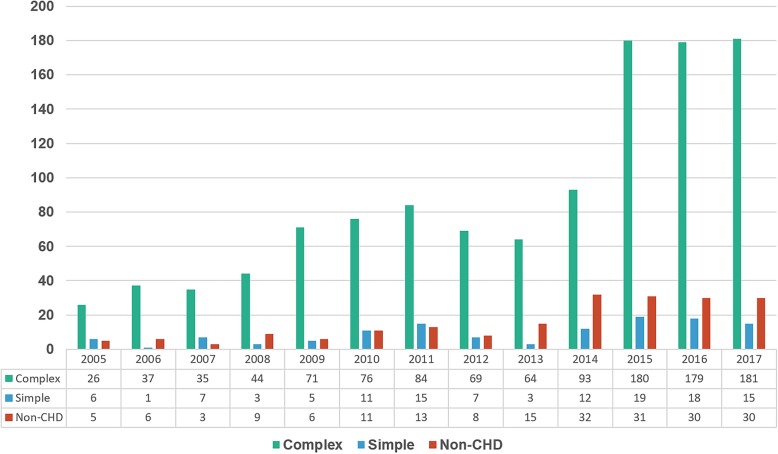
Annual Number of Adult Admissions by level of defect complexity. The ratio of patients with complex CHD did not change from 2005 to 2017 and it was from 70 to 80%

**Fig. 4:**
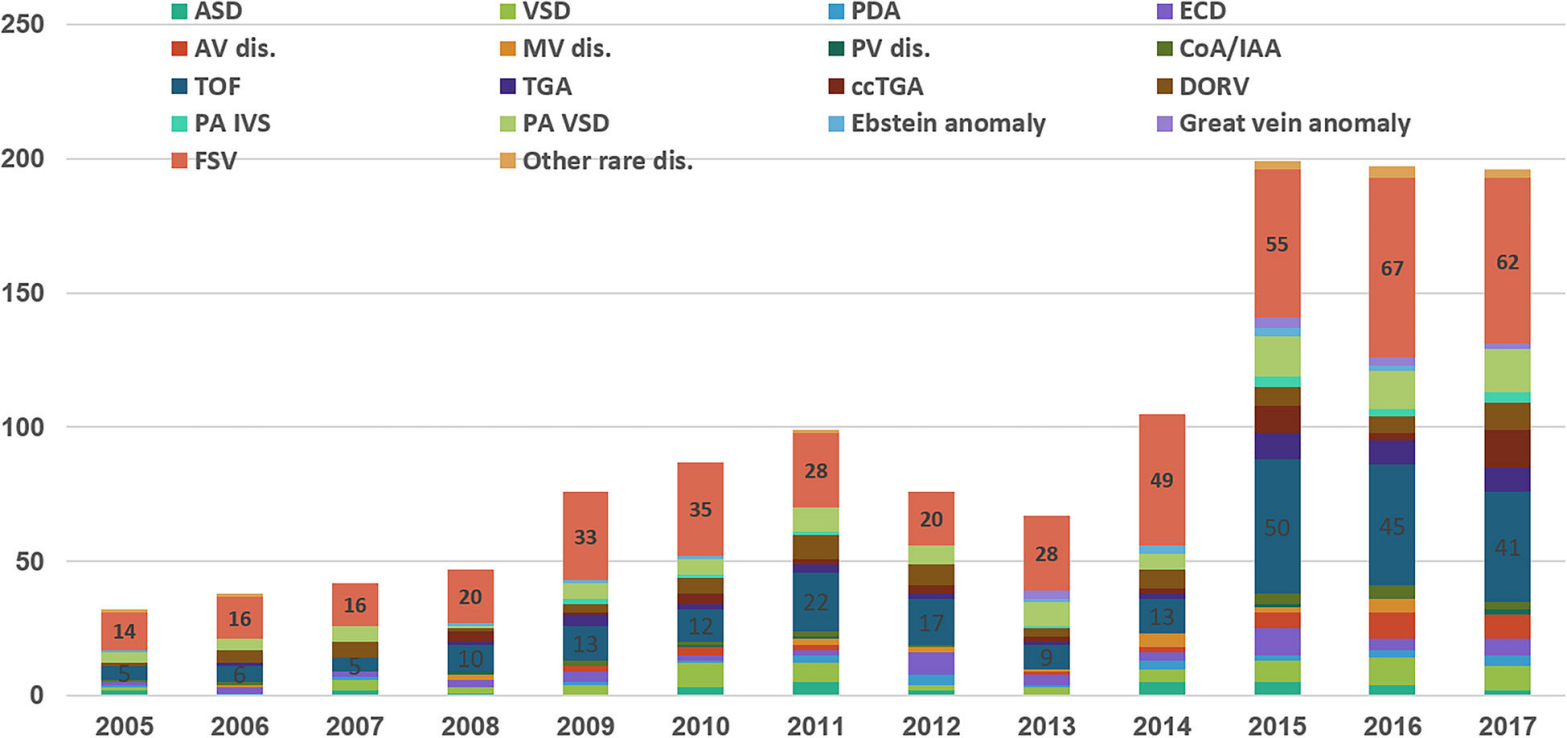
Annual Number of Admissions for Adults with congenital heart disease diagnoses. Functional single ventricle was the most common single complex diagnosis in all years and tetralogy of Fallot (TOF) was second

**Fig. 5:**
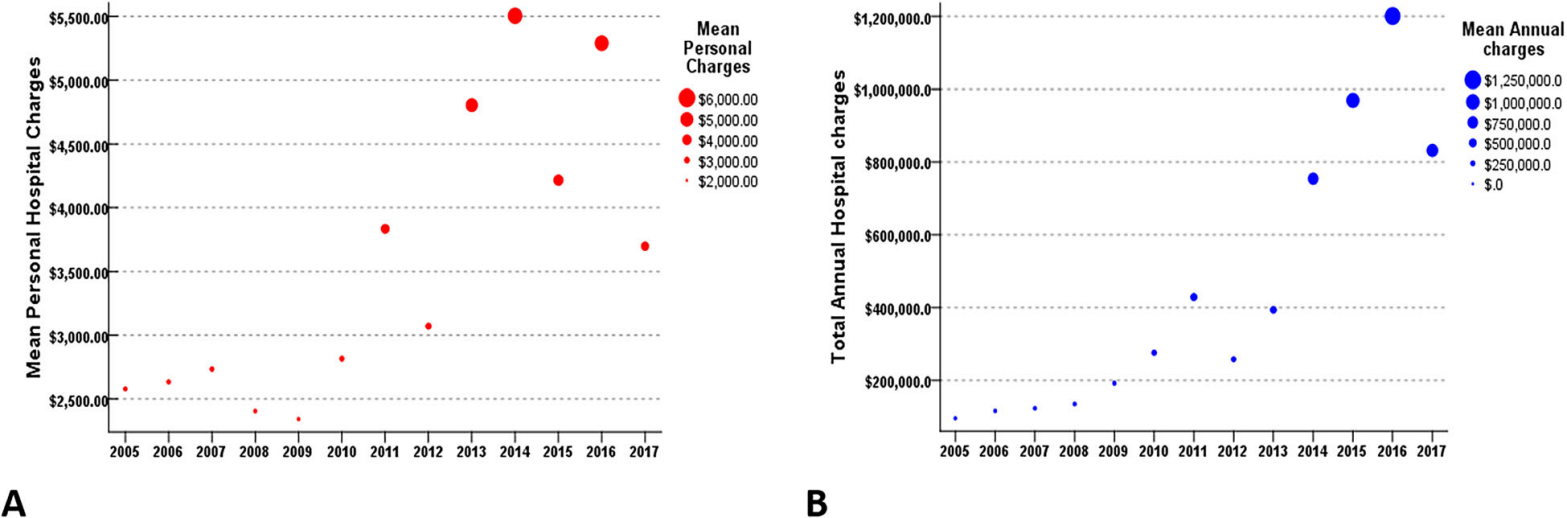
**a** Mean personal hospital charges and (**b**) Total annual hospital charges for admission of adults with congenital heart disease (US dollars). Mean personal hospital charges by admission of ACHD increased to around two times from 2005 to 2017. (from $2578.1 to $3697.0). Total annual hospital charges by ACHD markedly increased ten times (from $95,389.7 to $831,834.2)

The annual number of admissions for patients with complex CHD diagnoses is presented in Fig. 3. The ratio of patients with complex CHD did not change from 2005 to 2017 and it was from 70 to 80%. About 10% of patients each year were in the non-CHD group, most of whom had cardiomyopathy and coronary complication by Kawasaki disease. Functional single ventricle was the most common single complex diagnosis in all years and tetralogy of Fallot (TOF) was second (Fig. 4). Patients with complex CHD were younger on average than those with simple defects (26.4 ± 6.9 years vs. 31.0 ± 11.6 years, *p* < 0.000) and did not show a difference in sex ratio. Trends in specific selected diagnoses and procedures for ACHD admissions are presented in Table 1. Among them, arrhythmia and heart failure were common and infection-related complications were the most common diagnosis in the unclassified group according to comorbidities based on Elixhauser’s comprehensive set of comorbidities [8]. The mean hospital duration was 8.4 ± 17.0 days, and there was no significant difference from 2005 to 2017. (Table 1). The annual ICU admission percentage was around 5% and mean length of intensive care unit (ICU) stay was 8.4 ± 14.6 days. The absolute number of patients who underwent ICU care and duration of ICU stay increased significantly from 2005 to 2017 in recent years (Table 1). Mean personal hospital charges by admission of ACHD increased to around 1.4 times from 2005 to 2017. (from $2578.1 to $3697.0). Total annual hospital charges by ACHD markedly increased 8.7 times (from $95,389.7 to $831,834.2) (Table 1 and Fig. 5).

**Table 1:** Specific Diagnoses and Procedures Associated with Hospitalizations for ACHD Table 3 Frequency of Specific Diagnoses and Procedures Associated with Hospitalizations for ACHD. (₩1200 Korean dollar converted to $1 US dollar)

Year	2005	2006	2007	2008	2009	2010	2011	2012	2013	2014	2015	2016	2017
**Diagnoses**													
Arrhythmia	11	8	14	15	14	20	23	23	25	20	38	40	34
Heart failure	10	16	9	16	33	40	36	27	15	33	80	74	72
Pulmonary HTN	1	4	6	3	7	5	14	5	2	5	6	7	3
Endocarditis	0	1	1	1	0	0	1	0	0	3	3	4	3
CAD	0	0	0	0	2	3	1	0	1	3	3	5	9
**Procedures**													
Intervention	4	3	2	1	5	7	5	6	3	12	17	15	12
Device (pacemaker/ICD)	2	0	2	0	0	1	3	0	1	1	2	3	4
**Unclassified**	4	6	8	11	15	11	16	15	20	28	50	49	59
**Non-CHD**	5	6	3	9	6	11	13	8	15	32	31	30	30
**Total**	37	44	45	56	82	98	112	84	82	137	230	227	226
**Hospital duration (days)**	7.0	10.9	8.3	8.5	8.7	8.6	10.8	7.4	8.3	8.9	8.4	9.1	6.4
**ICU duration (days)**	2.3	2.0	3.0	2.0	2.8	4.7	4.2	3.0	8.3	13.5	11.3	13.5	8.6
**ICU admission number**	3/37 (8.1%)	4/44 (9.1%)	1/45 (2.2%)	2/56 (3.6%)	4/82 (4.9%)	7/98 (7.1%)	5/112 (4.5%)	2/84 (2.4%)	6/82 (7.3%)	8/137 (5.8%)	16/230 (7.0%)	11/227 (4.8%)	8/226 (3.5%)
**Mean Hospital Charges** **per patient (US$)**	$2578.1	$2633.0	$2733.4	$2403.8	$2340.8	$2815.5	$3833.7	$3071.4	$4805.9	$5505.5	$4216.1	$5289.7	$3697.0
**Total Hospital Charges** **per year (US$)**	$95,389.7	$115,854.1	$123,004.5	$134,615.2	$191,945.6	$275,918.0	$429,376.9	$257,995.9	$394,083.4	$754,252.4	$969,692.2	$1200,767.8	$831,834.2

*CAD* coronary artery disease, *CHD* congenital heart disease, *ICD* implantable cardioverter-defibrillator

## Discussion

The Korean Heart Foundation reported that operations for CHD have been performed since 1959 in South Korea, open heart surgery have been popular after the 1980s (http://www.heart.or.kr). About 4000 patients in a year underwent open heart surgery or percutaneous interventions for CHD after the 1980s, and their mortality is currently around 2%. For this reason, the mean age of adults with CHD is younger than other countries, especially the United States [9, 10]. Additionally, because our subject is only the admission of patients in the pediatric cardiology division, our patients’ mean age is younger than in the data of other countries. A unique point in our data is that the number of patients with simple defects is too low. This might be because our subject is only admitted patients in the pediatric cardiology division and our subject age is younger. In other reports, adults with CHD who have simple lesions might have complications at an older age than those with complex lesions [9].

Like research in other countries, our results showed increasing adults with CHD, and their number will be about 70,000 in South Korea by the year 2020 according to Kim’s report [5]. In a previous investigation [9] it was reported that this trend derived from improvements in care, increasingly sensitive diagnostic tools, and increasing awareness of the potential relationships between common diseases and CHD. In our country, surgical mortality for CHD was relatively pretty low nowadays and the quality of imaging techniques is relatively so high [11].

The growing population of adults with CHD requires efficient healthcare organizations. We need a multi-dimensional approach to defining the role of different healthcare professionals, improvement of communication channels, and certification or training programs for healthcare professionals including physicians, surgeons, and nurses [2, 12]. A follow-up program or guidelines for specific CHDs might be useful and help physicians with the long-term management of patients and early detection of various complications [2]. In our results, adults with CHD requiring ICU care and ICU stays have been increased with the increasing total adults with CHD. When adult patients with CHD undergo ICU care, knowledge of CHD might be critical, and the need for pediatric cardiologic intensivists is increasing. Because pediatric cardiologic intensivists usually have experience in post-operative periods, heart failure, mechanical cardiac support, and post-heart transplantation management, they can decide the optimal timing for mechanical cardiac support and heart transplantation [12].

In our results, the main causes of admission for adults with CHD were arrhythmia, heart failure, and single ventricle-related complications. This might be because our hospital is the oldest tertiary institution in South Korea with many patients who had complex CHDs and the ratio of complex CHDs is high. (Fig. 3 and Table 1).

As our results show, total hospital charges for adults with CHD are snowballing. As mentioned before, efficient healthcare for adults with CHD needs a multi-dimensional approach, effective communication, and professional training. With these efforts, healthcare charges for adults with CHD can be saved against a social background where life expectancy is increasing significantly in South Korea.

The principal limitation of this study is that we only analyzed admission cases in the pediatric cardiologic division. Therefore, adults with CHD from other departments with conditions such as metabolic syndrome and pregnancy were not included, although these conditions were not uncommon [13, 14]. The other limitation is that this result could not reflect true epidemiologic changes of South Korea, because our center is tertiary referral national center and many patients were referred far from our hospital. However, our data is useful for understanding changes in pediatric cardiology trends in one tertiary referral center. Additionally, our data has accuracy and completeness compared to other reports because we used diagnostic codes and reviewed medical records directly, in contrast with other reports that only used diagnostic codes [9, 15, 16]. Although our study was conducted retrospectively, it was difficult to conduct it prospectively on the subject of the study.

## Conclusion

Our study shows an increase in the number and rate of hospital admissions for adults with CHD, and their mean age has been increased every year. Main problems of adults with CHD were heart failure, arrhythmia and single ventricle-related complications. Because our subjects are relatively younger than in other countries, these trends will be strongly progressive. We need preparations for efficient healthcare for adults with CHD such as a multi-dimensional approach, effective communication, and professional training.

## Supplementary information

**Additional file 1: Supplement table.** Each diagnosis of adult patients with congenital heart disease was categorized as simple, complex, or unclassified based on the 32nd Bethesda Conference report [3].

## Data Availability

The datasets used and/or analyzed during the current study will be available from the corresponding author on reasonable request.

## Abbreviations

ACHD: Adults with congenital heart disease
ASD/PFO: Atrial septal defect/ patent foramen ovale
CHD: Congenital heart disease
ICD: International classification of diseases
ICU: Intensive care unit
PDA: Patent ductus arteriosus
TOF: Tetralogy of Fallot
