# Supplementary material for: Changes of hospitalization trend in the pediatric cardiology division of a single center by increasing adult with congenital heart disease
Source: BMC Cardiovasc Disord. 2020 May 15;20:227. doi: 10.1186/s12872-020-01511-3 (PMC7229598; doi:10.1186/s12872-020-01511-3)
Supplement: Supplementary file 1 — Additional file 1: Supplement table. Each diagnosis of adult patients with congenital heart disease was categorized as simple, complex, or unclassified based on the 32nd Bethesda Conference report [3]. [file 12872_2020_1511_MOESM1_ESM.doc]

**Supplement table.** Each diagnosis of adult patients with congenital heart disease was categorized as simple, complex, or unclassified based on the 32nd Bethesda Conference report [3].

1. Types of adult patients with simple congenital heart disease

| Native disease  Isolated congenital aortic valve disease  Isolated congenital mitral valve disease (e.g., except parachute valve, cleft leaflet)  Isolated patent foramen ovale or small atrial septal defect  Isolated small ventricular septal defect (no associated lesions)  Mild pulmonic stenosis  Repaired conditions  Previously ligated or occluded ductus arteriosus  Repaired secundum or sinus venosus atrial septal defect without residua  Repaired ventricular septal defect without residua |
| --- |

1. Types of adult patients with congenital heart disease of moderate severity

| Aorto-left ventricular fistulae  Anomalous pulmonary venous drainage, partial or total  Atrioventricular canal defects (partial or complete)  Coarctation of the aorta  Ebstein’s anomaly  Infundibular right ventricular outflow obstruction of significance  Ostium primum atrial septal defect  Patent ductus arteriosus (not closed)  Pulmonary valve regurgitation (moderate to severe)  Pulmonic valve stenosis (moderate to severe)  Sinus of Valsalva fistula/aneurysm  Sinus venosus atrial septal defect  Subvalvar or supravalvar aortic stenosis (except HOCM)  Tetralogy of Fallot  Ventricular septal defect with  Absent valve or valves  Aortic regurgitation  Coarctation of the aorta  Mitral disease  Right ventricular outflow tract obstruction  Straddling tricuspid/mitral valve  Subaortic stenosis |
| --- |

HOCM = hypertrophic obstructive cardiomyopathy

1. Types of adult patients with congenital heart disease of great severity

| Conduits, valved or nonvalved  Cyanotic congenital heart (all forms)  Double-outlet ventricle  Eisenmenger syndrome  Fontan procedure  Mitral atresia  Single ventricle (also called double inlet or outlet, common or primitive)  Pulmonary atresia (all forms)  Pulmonary vascular obstructive diseases  Transposition of the great arteries  Tricuspid atresia  Truncus arteriosus/hemitruncus  Other abnormalities of atrioventricular or ventriculoarterial connection  not included above (i.e., crisscross heart, isomerism, heterotaxy syndromes, ventricular inversion) |
| --- |
